# Meningococcal disease in Italy: public concern, media coverage and policy change

**DOI:** 10.1186/s12889-019-7426-5

**Published:** 2019-08-07

**Authors:** Loredana Covolo, Elia Croce, Marco Moneda, Elena Zanardini, Umberto Gelatti, Peter J. Schulz, Elisabetta Ceretti

**Affiliations:** 1 Department of Medical and Surgical Specialties, Radiological Sciences and Public Health - Unit of Hygiene, Epidemiology and Public Health, University of Brescia, Brescia, Italy; 2 Post-Graduate School of Public Health, University of Brescia, Brescia, Italy; 3 Institute of Communication and Health, Faculty of Communication Science, University of Lugano, Lugano, Switzerland

**Keywords:** Meningitis, Media coverage, Public health, Policy

## Abstract

**Background:**

Between 2015 and 2017 six deaths due to meningitis in the Lombardy Region, Northern Italy, caught the attention of media and increased concern among the population, with a consequent increase in demand for vaccination. Considering the evidence about the impact of media coverage of health issues on public behaviour, this paper investigates the trend of media coverage and internet searches regarding meningitis in the Lombardy Region.

**Methods:**

Content analysis of online articles published from January 2015 to May 2017 and analysis of Google Trends were carried out. A codebook was created in order to assess the content of each article analysed, based on six areas: article characteristics, information about meningococcal disease and vaccination, Local Health Authority activities, accuracy of information and tone of the message.

**Results:**

Both public interest and media attention peaked in December 2016 and January 2017, when the Lombardy Regional Authority changed its policy by offering co-payment to adults with a saving of 50%. The frequency of meningitis coverage decreased after the announcement of policy change. For example, articles containing new information on meningitis or meningococcal vaccine (76 to 48%, *p* = 0.01) and preventive recommendations (31% down to 10%, *p* = 0.006) decreased significantly. An alarmist tone appeared in 21% of pre-policy articles that decreased to 5% post-policy (*p* = 0.03).

**Conclusions:**

The findings suggest a role for the media in fostering public pressure towards health services and policy-makers. A collaboration between Public Health institutions and the media would be beneficial in order to improve communication with the public.

**Electronic supplementary material:**

The online version of this article (10.1186/s12889-019-7426-5) contains supplementary material, which is available to authorized users.

## Background

Meningococcal disease has always been a significant health problem worldwide, due to the related severe complications and high mortality rates [1]. From 2011 to 2016, approximately 150–200 cases of meningococcal meningitis occurred in Italy every year, with an annual peak in the winter months [2]. In contrast to the usual patterns of prevalence, the Tuscany Region reported a steep increase in cases of invasive meningococcal disease, from 12 and 16 cases in 2013 and 2014 respectively, to 43 cases in 2015 [3]. In August 2016, a girl died of meningitis during World Youth Day. These events triggered mass media interest, thus increasing people’s attention and public concern [4]. In fact, media is not only a reflection of popular opinion, but it can drive popular opinion as well also thanks to Web 2.0. The latter has made it possible for people to interact, share and discuss any information, stories or experiences. What happens is that it takes only a few stories with an emotional impact to contribute to risk perception, as extensively shown in online vaccination debate [5].

In early childhood, free vaccination is provided by the Italian National Health Service (NHS) against serogroup C and, since January 2017, against serogroup B, in order to eliminate regional differences, according to the new National Vaccine Plan 2017–2019 [6]. Meningococcal vaccinations were not provided to healthy adults for free by the NHS and was not mandatory for any kind of occupation. On 29th December 2016, the Regional Committee of Lombardy (Northern Italy) approved a new vaccination campaign against meningitis with a co-payment system for adults, starting 9th January 2017 [7]. The co-payment system offered the possibility for citizens to buy the vaccines at the same price paid by Region, i.e. 44 euro instead of 92 euro for Meningococcal Group ACWY, and 65 euro instead of 164 euro for Meningococcal Group B with a saving of around 50% [8].

Offering a new health protection service reflects a change in public health care policy. Such policy changes are a way for the government to respond to social problems that have newly developed, recently increased, or moved into the society’s focus of attention for other reasons. Policy changes can therefore be influenced according to the ‘outside initiative model’, which basically holds that society has to exert pressure on political decision makers to make them attend to problems. In this context, society refers to two entities, i.e., public opinion and the mass media [9].

If the ‘outside initiative model’ were true, public interest and media attention towards a problem should rise and remain elevated for some time, which would be essential to trigger and observe a policymaking response. After policy changes were implemented, public interest and media attention would either subside, if the public and media are satisfied with that policy change, or remain elevated, if they are not. The model is further complicated by the fact that public opinion and mass media need not behave in the same way and have a potential to influence one another [9].

The ‘outside initiative model’ is not the only way to conceptualise the influence processes between public opinion, mass media and policy makers. It is easy to imagine that when policymakers, are alerted to a problem, they produce a solution, which is then publicised and focuses media attention and/or public interest on the problem but only after a solution is found. Such models can be conceived similar to William F. Ogburn’s [10] concept of a cultural lag, which states that material change is more dynamic than the cultural reactions to it. For social problems and public policies, such concepts can be called communication lag models, which form the basic alternatives to the outside initiative model. In both models, there is ample space for dysfunctional or irrational influences.

A similar model that explains these dynamics is called the ‘mobilisation model’ [9], that is, when policy-makers take the initiative to put an issue on the political agenda and then attempt to gain support for the issue by raising public attention and involving the media.

This study aimed to describe the dynamics of the decision made by the Regional Committee of Lombardy on 29th December 2016, regarding co-payment meningococcal vaccination for adults [7] and try to understand if the mobilization model or outside initiative model better fit the data. The public interest and media attention surrounding meningococcal disease were measured and quantitative analysis of media content and coverage, which may have affected the process, was carried out.

## Methods

The study covers the period from January 2015 until May 2017. Public interest was assessed using Google Trends and media attention was measured through an analysis of articles from leading newspapers.

**Fig. 1:**
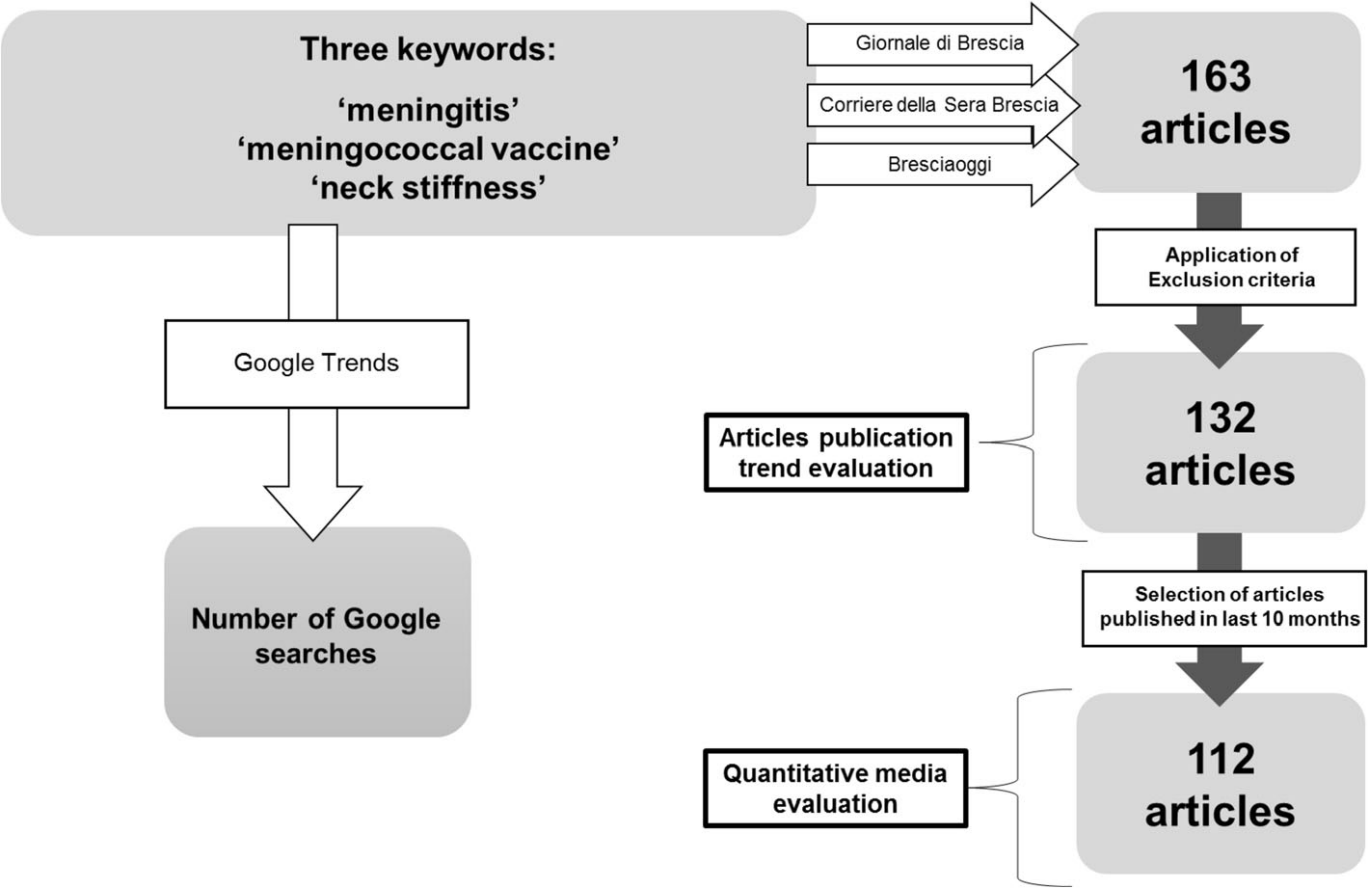
Flowchart of the study process

Google Trends is an online tool that measures how often one or more keywords are searched over a specific time period [11]. We used the Italian equivalents to meningitis, meningococcal vaccine and neck stiffness as keywords (Fig. 1), limiting the research to Lombardy Region. Google Trends does not show the overall number of searches, but the percentages, with the highest value in the time series being defined as 100%. A peak was considered as an increase of more than 25% of searches in less than 2 months.

Media attention towards meningitis was measured by the number of articles featured in the online version of three main local newspapers from Brescia (*Bresciaoggi, Corriere della Sera di Brescia and Giornale di Brescia*). We used the Italian equivalents to meningitis as keyword in each newspaper. We only selected articles on meningitis cases or vaccinations, excluding videos and letters to the editor. The latter have been excluded because they represented personal comments rather than a mere summary of facts.

A quantitative content analysis of articles published, in the last 10 months of the study period, was performed (Fig. 1). We split this period in two: the 5 months before (1st August–December, 2016) and after the policy change (30th December–May 2017).

This period corresponded to the major distribution of articles focused on themes related to policy change. We chose a 5 months period before 29th December to understand what influence policy change and a same period after this date to understand the consequences of the policy change.

We created a codebook (Additional file 1), according to the method proposed by Riffe et al. [12]. In order to analyse the content of each online article. The codebook included 20 items, summarised in six main areas:

- Article characteristics (type of the article, main theme, presence of new information, etc);
- Information about the meningococcal disease (description of cases, geographical location, signs and symptoms of meningitis, prophylaxis recommendations);
- Information about the meningococcal vaccine (types of available vaccines, costs, risks and benefits and vaccination centre contacts);
- Local Health Authority (LHA) activity (link to institutional sources and description of LHA interventions);
- Accuracy of information: inaccurate information, if mistakes were present; misinformation, if sources were not used correctly, and incorrect conclusions according to scientific evidence were present.
- Message tone, defining it as ‘alarmist’ if there was excessive angst regarding meningitis and/or an emphasis of facts (e.g. using words such as ‘anxiety’, ‘terror’, ‘scare’ or ‘panic’), or ‘reassuring’ if the necessity to avoid panic was emphasised with sentences such as ‘there is no emergency’, ‘there is no epidemic’, ‘the situation is no different to previous years’, and ‘neutral’ if information was unbiased and without any sort of emphasis.

The main theme was coded into five categories (disease description, vaccination, policy, meningitis case reporting, other). All other variables were coded as present or absent. ‘New information’ was defined as the first time a specific topic appeared in a journal, during the study period.

The selected articles were categorised and analysed independently by two researchers (E.C., M.M.). The agreement on classification of articles reached the 95% and discordance were solved by a third party researcher (L.C.).

Chi-square and Fisher’s exact tests were performed for the analysis of contingency tables.

## Results

The search terms yielded a total of 163 articles, but only 132 (81%) of them fulfilled the inclusion criteria: 53 (40%) from *Bresciaoggi*, 28 (21%) from *Corriere della Sera di Brescia* and 51 (39%) from *Giornale di Brescia*. Trend analysis of newspaper attention was based on these 132 articles.

**Fig. 2:**
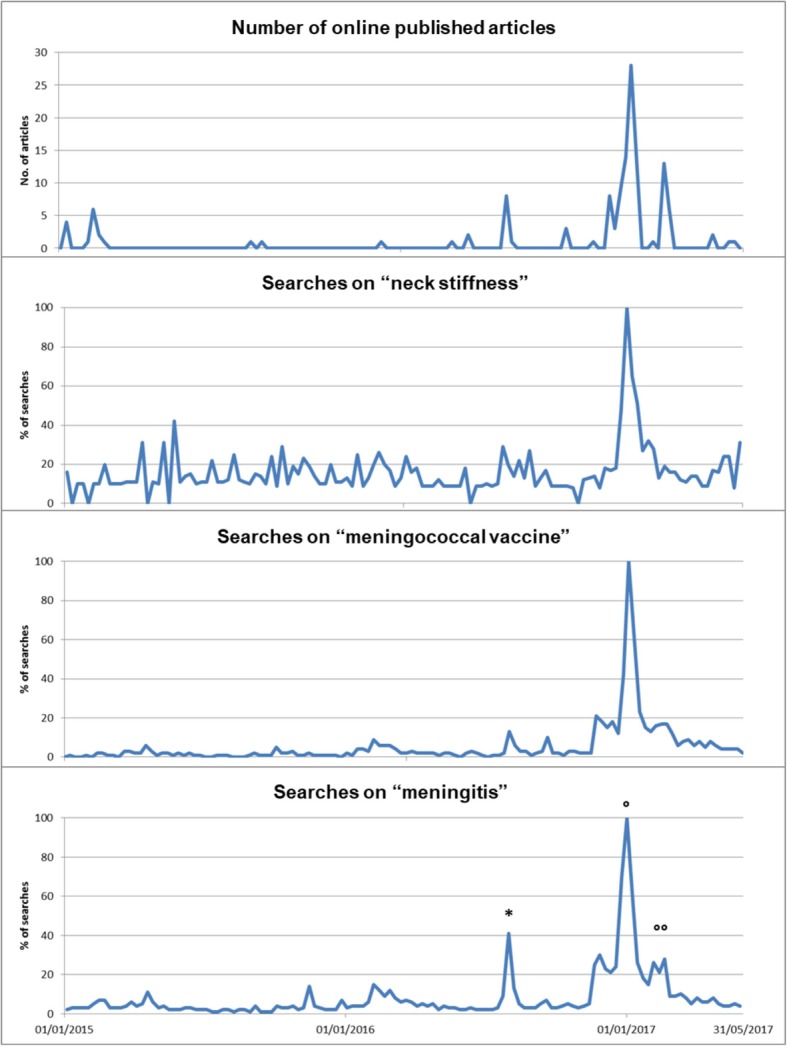
Total number of online published articles and Google searches on keywords in the study period. *case of Roman girl died in Vienna (August 2016); °three meningitis’ deaths in Lombardy (November 2016 – January 2017) and policy change (29-12-2016); °° three meningitis’ deaths in Lombardy (February 2017)

Both public interest towards meningitis and newspaper attention had briefly risen in August 2016 (one case of death due to meningitis). An obvious peak occurred in December 2016 and January 2017, when three cases of meningitis were recorded in Lombardy, which resulted in changes to regional policy to offer co-payment to adults (Fig. 2). Media attention on the subject endured into February 2017 but without a corresponding peak in public interest.

Excluding the increase in August 2016, searches for ‘meningococcal vaccine’ and ‘meningitis’ stood at about 4–5% of the maximum, observed in early January 2017 (set by Google trends as 100%). Prior to the peak, the number of searches for the keyword ‘neck stiffness’ stood at about 15% of the relative maximum peak. Newspaper attention amounted to 1.25 articles per month before the peak, while 34 articles were published during the peak, in December 2016, that is, about 25 times higher than normal. Overall, the peaks for Google searches using the three keywords and the number of online articles are comparable.

**Fig. 3:**
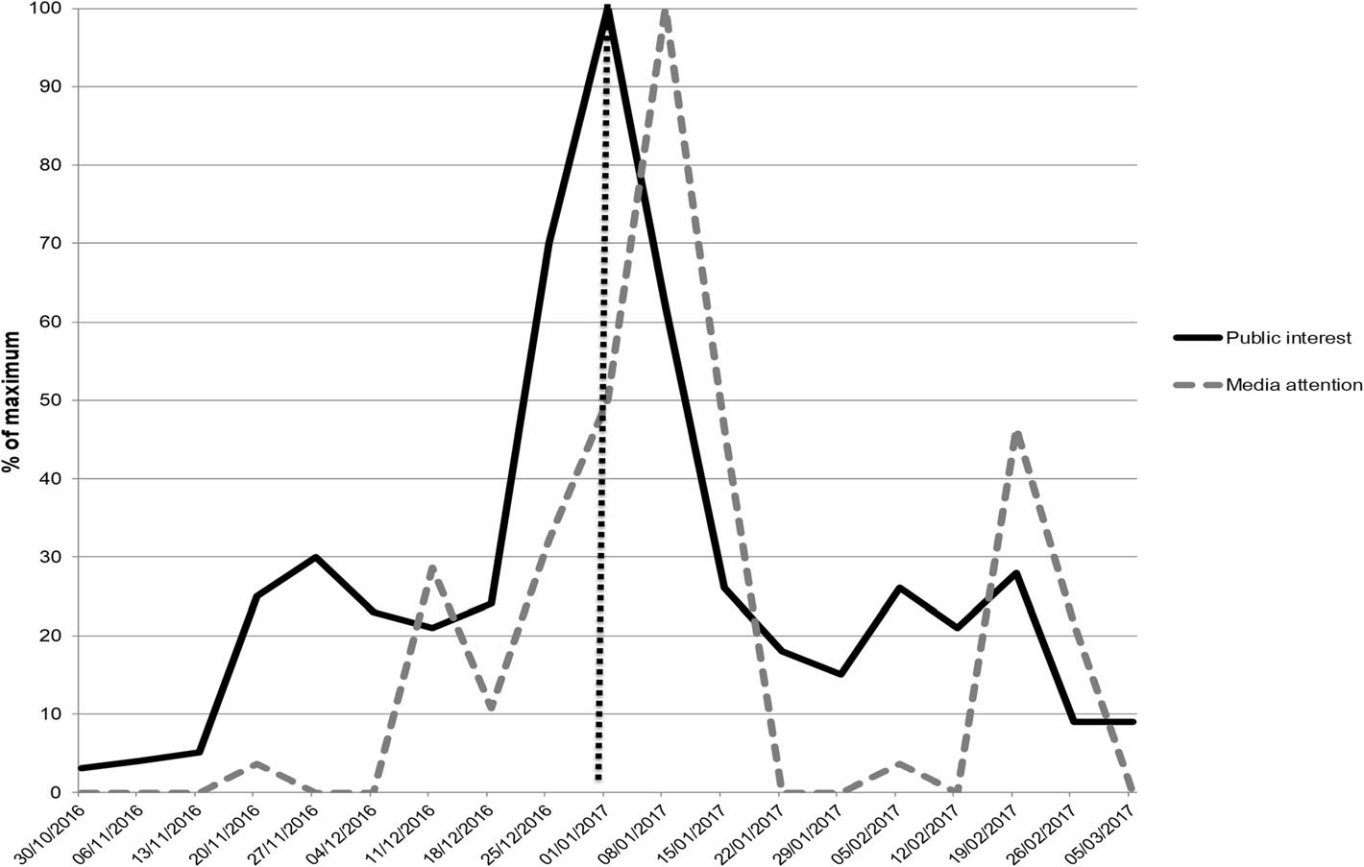
Total number of online published articles (“media attention”) and Google searches on keywords (“public interest”) before and after 29th December 2016, date of new vaccination campaign against meningitis approval

Figure 3 shows the peak months of December 2016 to February 2017 in detail. We noted an increase in public interest from 18th December 2016 to 1st January 2017, coded as maximum concern. Media attention showed a similar trend but with a lag of 1 week. Another rise in media attention began on 12th February 2017 and reached about 45% of the previous peak on 19th February 2017, descending to 0 until 5th March 2017. This rise had no corresponding peaks in public interest.

A quantitative content analysis of articles published in the last 10 months of the study period was performed. Of the 112 articles (Additional file 2), 29 (26%) were published before and 83 (74%) after the vaccination policy change). Thirty-seven articles (33%) were published in the *Bresciaoggi*, 26 (23%) in the *Corriere della Sera di Brescia* and 49 (44%) in the *Giornale di Brescia*.

Table 1 shows the results of quantitative evaluation. With regard to the main theme, there was an increase in articles on vaccination after the policy change (4% up to 28%, *p* = 0.007) and a decrease in meningitis case reporting was observed (59% down to 30%), *p* = 0.002).

**Table 1:** Quantitative media evaluation of online articles published on local newspapers in the study period

Area	Items	Total (*n* = 112) No. (%)	Pre policy (*n* = 29) No. (%)	Post policy (*n* = 83) No. (%)	*P* value
Article characteristics	Type of article				
	Editorials	26 (23)	7 (24)	19 (23)	ns
	General news	86 (77)	22 (76)	64 (77)	ns
	Main theme				
	Disease description	7 (6)	3 (10)	4 (5)	ns
	Vaccination	24 (21)	1 (4)	23 (28)	0.007^a^
	Policy	12 (11)	3 (10)	9 (11)	ns^a^
	Meningitis case reporting	42 (38)	17 (59)	25 (30)	0.002
	Other	27 (24)	5 (17)	22 (27)	ns
	New information	62 (55)	22 (76)	40 (48)	0.01
	Mention of institutions or literature	13 (12)	3 (10)	10 (12)	ns^a^
	Health information provided by professionals	9 (8)	4 (14)	5 (6)	ns^a^
Information about meningococcal disease	Case description	43 (38)	11 (37)	32 (39)	ns
	Signs and symptoms	16 (14)	11 (38)	5 (6)	< 0.001^a^
	Long term effects	3 (3)	1 (3)	2 (2)	ns^a^
	Preventive recommendations	17 (15)	9 (31)	8 (10)	0.006
Information about meningococcal vaccine	Available vaccines	28 (25)	6 (21)	22 (27)	ns
	Cost of vaccination	23 (21)	4 (14)	19 (23)	ns^a^
	Risks and/or benefits	5 (4)	2 (7)	3 (4)	ns^a^
	Vaccination centre’s contacts	18 (16)	1 (3)	17 (20)	0.038
LHA activity	Link to institutional sources	5 (4)	1 (3)	4 (5)	ns^a^
	Description of LHA interventions	70 (63)	19 (66)	51 (61)	ns
Accuracy of information	Inaccurate information	7 (6)	1 (3)	6 (7)	ns^a^
	Misinformation	1 (0.9)	0	1 (1.2)	–
Tone	Reassuring	19 (17)	7 (24)	12 (15)	ns
	Neutral	83 (74)	16 (55)	67 (81)	0.007
	Alarmist	10 (9)	6 (21)	4 (5)	0.03^a^

^a^ Fisher’s exact test

*LHA* Local Health Authority, *ns* not significant

The frequency of meningitis coverage decreased after the announcement of policy change. In particular, this is statistically significant for articles containing new information on meningitis or the meningococcal vaccine (76% down to 48%, *p* = 0.01), signs and symptoms of meningitis (38% down to 6% of articles, *p* < 0.001), preventive recommendations (31% down to 10%, *p* = 0.006). This trend is similar for articles on information provided by health professionals, albeit not statistically significant (14% down to 6%, *p* > 0.05). The only significant increase occurred with regards to information about the meningococcal vaccine, that is, for vaccination centre contact information (3% up to 20%, *p* = 0.038). An alarmist tone appeared in 21% of pre-policy articles that decreased to 5% post-policy (*p* = 0.03). Three out of six articles, with an alarmist tone, were published on 11th December 2016, this can be considered a small peak prior to policy change (Fig. 3). Although of no statistical significance, there was a decrease in the amount of articles with a reassuring tone after the policy change (24% down to 15%, *p* > 0.05).

## Discussion

In the last 10 months of the study period, six deaths due to meningitis were recorded in Lombardy, two in the 5 months before policy change and four in the 5 months thereafter [13]. Media attention towards meningitis and public interest both surged in the winter of 2016–2017. The temporal coincidence of the peaks in media attention and public interest with policy change suggest a relationship among the three aspects.

The findings of this study seem to support the idea of the outside initiative model [9], when considering the public interest sourced from Google Trend. Prior to the policy change, there was an increase in public interest that might suggest public opinion was pressuring policy makers for some time until they took action. After all, there are also some articles in which policy makers stated that the decision to offer co-payment meningococcal vaccination to adults was taken due to increasing public concern for meningitis [14–16].

When considering media attention, a communication lag model or mobilisation model seem to fit the results more appropriately. In other words, the new regulation may have created some echo with newspapers which lasted for a few weeks, suggesting some sort of support from the media to policy-makers. About two weeks after the policy change, a decrease both in public interest and media attention was observed.

The strict relationship between the media coverage of health issues and public attitudes and behaviours has been evidenced in other contexts [17]. When analysing the content of articles, it was interesting to note that an alarmist tone was prevalent in 21% of pre-policy articles and decreased significantly to 5% post-policy (*p* = 0.03). In particular, as shown in Fig. 3, it is possible that public interest was triggered by the articles published on 11th December 2016. It should be noted that all of these articles had an alarmist tone. This could explain the increase in public interest, and probably concern that could have led to pressure on policy-makers. However, there was also a similar percentage of articles with a reassuring tone (24%) underlining the lack of a health emergency. Ultimately, it was evident that events causing fear or concern affect people more than positive or reassuring news [18, 19].

Previous studies found that an alarmist tone can ignite fear in readers [20] increasing the perceived severity and vulnerability of a disease [21]. At times it could lead to unnecessary measures being taken such as the extended closure of schools due to two cases of meningitis, despite public health recommendations [22], or inappropriate behaviour such as the increase of antibiotic use similar to the case of H1N1 influenza epidemic in 2010 [23].

The observed decrease in an alarmist tone, after the policy change, could be due to several factors, for example, the reduction in death announcements and the policy change itself.

Following the Lombardy policy change announcement, the results showed a reduction in new information regarding meningitis, particularly in terms of signs and symptoms and prophylaxis recommendations to avoid infection. At the same time, the information on the meningococcal vaccine increased, particularly on information about access to vaccination services. This may reflect an interest to inform the public on vaccines, their costs and availability rather than on the disease characteristics, as if media attention shifted from problem to solution.

Nevertheless, the media could also prove to be a helpful partner of public health institutions in communicating correct recommendations for disease management [21, 24]. Essentially, as also stated by other authors [25], a cooperation between journalists and public health communicators would be beneficial in order to deliver scientific and objective information to the population. On one side, the journalist requires the technical knowledge of public health specialists in order to guarantee accuracy and, on the other side, the public health specialists require the expertise of journalists in reporting health news.

This is particularly important to avoid the negative consequences of a ‘media epidemic’, as a useless demand overloads health systems. According to the epidemiological data published in January 2017 [26], the number of meningitis cases that occurred in the Lombardy Region in 2016 (*n* = 30) was comparable to that of cases recorded in 2015 (*n* = 34). However, the news regarding an abnormal increase in the amount of meningitis cases in the central region of Italy (Tuscany Region) occurred in January 2015, in addition to the six meningitis-related deaths publicised in the Lombardy Region contributed to an apparent increase in the number of cases, as if it was an epidemic disease. Public concern has translated into high demand of vaccinations. Particularly in Brescia, as reported by a local online newspaper [27], demand from citizens developed so fast that, in June 2017, the earliest available appointment to perform a meningitis vaccination was June 2019. Evidently, as it was not a real health emergency, the LHA did not implement a vaccination service. It should be noted that long waiting lists, in a public-perceived health emergency situation, can lead to a decline in the reputation of health authorities. In fact, in an interview conducted by a national newspaper [28], the Regional Councillor for Health revealed that approximately 30% of people missed their scheduled appointments, suggesting that people reduce their anxiety by arranging a vaccination appointment, and then, once the anxiety has settled, they forget to show up. This behaviour could risk preventing or delaying vaccination to those who really needed it.

This is an example of what happens when fear of disease predominates. In fact, the high demand of vaccinations observed in this scenario sounds unusual at a time when, also at international level, health professionals are questioning how to deal with the vaccine hesitancy [29]; a time in which the negative trends of paediatric coverage levels (under 95%) lead to the introduction of compulsory vaccination in Italy on July 2017 for ten infectious diseases [30] and a time in which the coverage of flu vaccination, important not only for elderly, is just around 15% among general population [31].

This study has several limitations, the results of the study could appear limited considering that the research has focused only on the local media of a city in Northern Italy. However, Brescia is one of the most populous provinces in Lombardy [32], a Region characterised by the highest estimated cases of meningococcal disease in Italy (46 cases out of 232, 20% in 2016) [2]. In 2016, the incidence rate of meningitis in the province of Brescia was 0.5/100.000 [33], similar to that of the Lombardy Region in the same year (0.4/100.000) [2]. Moreover, the dynamics of the media and public response, which we observed, towards the meningitis disease outbreak, has also been witnessed in other countries, in cases such as a meningitis or swine flu outbreak [20–24].

Only Google was used in terms of a web search engine, however, was the most frequently used search engine in Italy in the study period, representing 94% of web searches [34]. A limitation regarding Google Trends is that it does not show the number of web searches, but rather their percentage in relation to the maximum amount of searches. Even if it gives an estimation, it is a good tool to obtain real-time data on how people are using Google. Furthermore, the problem is that it is not possible to extrapolate the characteristics of those people surfing the web for health information. It should be highlighted that web users are not representative of the entire population, albeit a large percentage of population (59%) use the internet to search for health information [35].

## Conclusions

This is a good, localised example of what seems to be representative of the international context for many countries i.e. media and health professional scare-mongering. The situation that occurred in the Lombardy Region and, in particular, in Brescia highlighted the consequences that arose from a misinterpretation of the population about a health event, in terms of policy makers’ actions and public behaviour.

This event ultimately drives us to reflect on how difficult, in an age where information is quickly transmitted via the web, it is to control the spread of fear in a population. In fact, the policy changed due to a public-perceived health risk, at the media level. It should be noted that before the policy change, only two meningitis-related deaths had occurred and after the policy change, public interest decreased, even though another four deaths due to meningitis occurred. At the same time only 5% of articles had an alarmist tone.

This study has confirmed the important role of a media echo when dealing with public health issues. For this reason, as clearly stated by other studies [17, 24, 25] a collaboration between journalists and public health institutions would be strategic in order to improve communication with a population.

## Additional files

Additional file 1: Codebook. This file provides the codebook used to analyse the content of online articles. (PDF 313 kb)

Additional file 2: Links’ list of online articles published from August 2016 to May 2017. This file provides the list of URL related to 112 articles on which quantitative content analysis was performed. (DOCX 29 kb)

## Data Availability

The datasets used and/or analysed during the current study are available from the corresponding author on reasonable request.

## Abbreviation

LHA: Local Health Authority

## References

[1] Meningococcal meningitis. Fact sheet WHO reviewed January 2018. https://www.who.int/en/news-room/fact-sheets/detail/meningococcal-meningitis. Accessed 5 Aug 2019.

[2] Surveillance data for invasive bacterial diseases updated to 3 April 2017. National Institute of Health. http://www.iss.it/binary/mabi/cont/Report_MBI_20170403_finale.pdf. Accessed 18 Jan 2018.

[3] Stefanelli P, Miglietta A, Pezzotti P, Fazio C, Neri A, Vacca P (2016). Increased incidence of invasive meningococcal disease of serogroup C / clonal complex 11, Tuscany, Italy, 2015 to 2016. Eur Commun Dis Bull.

[4] Il giornale di Brescia. Meningite alla Gmg, morta una 19enne romana. «Evitare allarmismi». 2016 Aug 2. https://www.giornaledibrescia.it/italia-ed-estero/meningite-alla-gmg-morta-una-19enne-romana-evitare-allarmismi-1.3108756. Accessed 23 July 2019.

[5] Witteman HO, Zikmund-Fisher BJ (2012). The defining characteristics of web 2.0 and their potential influence in the online vaccination debate. Vaccine.

[6] Piano Nazionale Vaccini 2017–19. http://www.salute.gov.it/imgs/C_17_pubblicazioni_2571_allegato.pdf. Accessed 1 Oct 2017.

[7] Regione Lombardia Direzione Generale Welfare. Attivazione dell’offerta in copagamento in Regione Lombardia per la Prevenzione delle malattie invasive batteriche da meningococco. Decree number 14030 del 29/12/2016. https://www.ats-brescia.it/media/documenti/prevenzione_salute/decreto140302016_12753_13713.pdf. Accessed 1 Oct 2017.

[8] Regione Lombardia. ASST Spedali Civili. http://www.asst-spedalicivili.it/servizi/Menu/dinamica.aspx?idSezione=38862&idArea=38976&idCat=39533&ID=42311&TipoElemento=null. Accessed 14 Jun 2019.

[9] Lelieveldt H, Princen S. Agenda setting. In: Lelieveldt H, Princen S, editors. The Politics of the European Union. 2nd ed. Cambrige: Cambrige University Press; 2015. p. 205–11.

[10] Ogburn WF (1922). Social change: with respect to culture and original nature.

[11] Google Trends: https://www.thinkwithgoogle.com/tools/google-trends.html. Accessed 1 Oct 2017.

[12] Riffe D, Lacy S, Fico F (2005). Analyzing media messages: using quantitative content analysis in research.

[13] Bettoni S. Meningite mortale: casi in aumento. Gallera: «Nessuna emergenza». Corriere della Sera 2017 Nov 4: https://milano.corriere.it/notizie/cronaca/17_novembre_04/meningite-mortale-casi-aumento-gallera-nessuna-emergenza-d61c1c1a-c12c-11e7-b5e5-1f34efbbc6b1.shtml?refresh_ce-cp. Accessed 5 Aug 2019.

[14] Giornale di Brescia. Meningite, vaccino a costo ridotto da gennaio in Lombardia. https://www.giornaledibrescia.it/italia-ed-estero/meningite-vaccino-a-costo-ridotto-da-gennaio-in-lombardia-1.3140193. Accessed 28 Feb 2018.

[15] Bresciaoggi*.* Meningite, nessun rischio. http://www.bresciaoggi.it/territori/citt%C3%A0/meningite-nessun-rischio-1.5373313 Accessed 28 Feb 2018.

[16] Bresciaoggi. Meningite, parte la campagna regionale per la vaccinazione. http://www.bresciaoggi.it/territori/citt%C3%A0/meningite-parte-la-campagna-regionale-per-la-vaccinazione-1.5393432 Accessed 28 Feb 2018.

[17] Harrabin R, Coote A, Allen J (2003). Executive summary. Health in The News. Risk, Reporting And Media Influence.

[18] Tannenbaum MB, Hepler J, Zimmerman RS, Saul L, Jacobs S, Wilson K, Albarracín D (2015). Appealing to fear: a meta-analysis of fear appeal effectiveness and theories. Psychol Bull.

[19] Covolo L, Ceretti E, Passeri C, Boletti M, Gelatti U (2017). What arguments on vaccinations run through YouTube videos in Italy? A content analysis. Hum Vaccin Immunother.

[20] Chang C (2012). News coverage of health-related issues and its impacts on perceptions: Taiwan as an example. Health Commun.

[21] Goodall C, Sabo J, Cline R, Egbert N (2012). Threat, efficacy, and uncertainty in the first 5 months of national print and electronic news coverage of the H1N1 virus. J Health Commun.

[22] Ardern K, Bowler S, Hussey RMRC (1999). Managing meningococcal disease case clusters: art or science?. J Epidemiol Community Health.

[23] Bernier A, Ligier C, Guillemot D, Watier L (2013). Did media attention of the 2009 a(H1N1) influenza epidemic increase outpatient antibiotic use in France?: a time-series analysis. PLoS One.

[24] Singleton CD, Fey R, Appleby C (2000). Media management of a community outbreak of meningococcal meningitis. Commun Dis Public Health.

[25] Leask J, Hooker C, King C (2010). Media coverage of health issues and how to work more effectively with journalists: a qualitative study. BMC Public Health.

[26] National surveillance of invasive bacterial diseases updated at 2 Jan 2017. National Institute of Health: http://www.epicentro.iss.it/problemi/meningiti/EpidemiaMediatica.asp. Accessed 1 Oct 2017.

[27] Piaterra ML and Chiari G. Le vaccinazioni volano, l’attesa oltre 2anni. Bresciaoggi 2017 Feb 22: http://www.bresciaoggi.it/territori/le-vaccinazioni-volano-l-attesa-oltre-2anni-1.5511800. Accessed 1 Oct 2017.

[28] Bettoni S and Ravizza S. Meningite e vaccini, liste bloccate: «Corsia preferenziale per i bambini». Corriere della Sera 2017 Feb 22: http://milano.corriere.it/notizie/cronaca/17_febbraio_22/meningite-vaccini-liste-d-attesa-bloccate-una-corsia-preferenziale-bambini-5ea433f8-f8c7-11e6-ae6b-f2dcdeebb2b6.shtml. Accessed 17 Jun 2017.

[29] Godlee F (2019). What should we do about vaccine hesitancy?. BMJ.

[30] Legge n.119/17. Disposizioni urgenti in materia di prevenzione vaccinale. Available at: http://www.gazzettaufficiale.it/eli/id/2017/08/5/17G00132/sg. Accessed 14 Jun 2019.

[31] National Institute of Health. Influenza vaccination covers in Italy. Available at: https://www.epicentro.iss.it/influenza/coperture-vaccinali. Accessed 14 Jun 2019.

[32] ISTAT - Italian national statistical institute: http://demo.istat.it/pop2017/index.html. Accessed 30 Jan 2018.

[33] Report 2016 sulle attività di prevenzione e promozione della salute dell’ATS di Brescia. https://www.ats-brescia.it/media/documenti/pubblicazioni/2017/INTERATTIVO_300dpi.pdf. Accessed 30 Jan 2018.

[34] StatCounter. http://gs.statcounter.com/search-engine-market-share/all/italy/#monthly-201501-201701-bar. Accessed 17 May 2017.

[35] Flash Eurobarometer 404. European citizens' digital health literacy; 2014: http://observgo.uquebec.ca/observgo/fichiers/42526_res1.pdf Accessed 29 Jan 2018.

