# Supplementary material for: Meningococcal disease in Italy: public concern, media coverage and policy change
Source: BMC Public Health. 2019 Aug 7;19:1061. doi: 10.1186/s12889-019-7426-5 (PMC6686541; doi:10.1186/s12889-019-7426-5)
Supplement: Supplementary file 1 — Codebook. This file provides the codebook used to analyse the content of online articles. (PDF 313 kb) [file 12889_2019_7426_MOESM1_ESM.pdf]

Additional file 1: CODEBOOK

1. Article characteristics

1.1 Type of article:

- Editorials (opinion article or press release): any story that offers a first person opinion or is a stated opinion of the newspaper.
- General news (news article): any story that emphasize facts of a recent event.

1.2 Theme of the article (cross the best one with a X):

Most stories are complex and therefore involve more than one theme; you need to decide which theme is most prominent. The questions to answer are: what is the story really about? What is the ***main point*** *of the story? Focus on why this story is in the paper at all.*

A. Disease description (general description of meningitidis, epidemiology, symptoms, prevention)
B. Vaccination (description of anti-meningococcal vaccine, how to access the vaccination service, costs, waiting time)
C. Policy (communication by health institution on prevention and survelliance procedures)
D. Meningitidis case reporting (news of meningitis'case, death or disease's course)
E. Other (the article can not be categorized in previous themes and meningitis is not the main topic)

1.3 Primary/dominant intent of the message (write it using three keywords):

________________ ________________ ________________

1.4 New information (the article adds something new to information, regarding meningitis or meningococcal vaccine, contained in previous articles):

- Yes
- No

1.5 Presence of at least one mention of institutions or literature in the entire article:

- Yes
- No

1.6 Health information regarding meningitis or meningococcal vaccine are provided by health professionals (doctors, nurses, etc):

- Yes
- No

2. Information about meningococcal disease (included cases)

2.1 Case description is clear and complete:

Description clear and complete means it contains both the following information: geographical location and responsible bacteria.
Remember to note the following information:
- *Geographical location: B=brescia, L=lombardia, I=italy*
- *Responsible bacteria: M=meningococcus, P=pneumococcus, O=other*

- Yes
- No

2.2 Article contains information about signs and sympthoms of meningitis:
The article contains at least 2 of the following signs and sympthoms: headache; altered mental status; fever; neck stiffness.

- Yes
- No

2.3 Article contains information about long-term effects of meningitis:

- Yes
- No

2.4 Article contains information about preventive recommendations:

- Yes
- No

3. Information about meningococcal vaccine

3.1 Article contains information about different types of available vaccines:

- Yes
- No

3.2 Article contains information about which subjects can access to free or copayment vaccine and/or regarding vaccine's cost:

- Yes
- No

3.3 Article contains information about risks and/or benefits of meningococcal vaccine:
Remember to note down if the article talks just about benefits (b) , just about risks (r) or both (br): ________________

- Yes
- No

3.4 Article contains vaccination centre contact details (at least phone number, address or e-mail address):

- Yes

- No

4. Local Health Authorities activities

4.1 Article contains links to institutional sources, institutional websites or quote to institutional sources:

- Yes
- No

4.2 Article describes Local Health Authorities interventions or actions to manage meningococcal disease incident cases:

- Yes
- No

5. Accuracy of information

5.1 Article contains not correct information:

- Inaccurate information (presence of mistakes)
- Misinformation (sources are not used in a correct manner and incorrect conclusions according to scientific evidence are present)
- No

6. Tone of the message

6.1 The tone of the message is:

- “neutral” (information are unbiased and without any sort of emphasis)
- “alarmist” (there is an excessive apprehension about meningitis and/or an emphasis of facts, e.g. using words as “anxiety”, “scare”, “panic” or “drama” both in the title and in the text)
- ”reassuring” (the aim is to avoid panic so there are sentences as “there is no emergence” , “there is no epidemic”, “The situation is not different from previous years”)
