# Supplementary material for: Meningococcal disease in Italy: public concern, media coverage and policy change
Source: BMC Public Health. 2019 Aug 7;19:1061. doi: 10.1186/s12889-019-7426-5 (PMC6686541; doi:10.1186/s12889-019-7426-5)
Supplement: Supplementary file 2 — Links’ list of online articles published from August 2016 to May 2017. This file provides the list of URL related to 112 articles on which quantitative content analysis was performed. (DOCX 29 kb) [file 12889_2019_7426_MOESM2_ESM.docx]

**Additional file 2**: Links’ list of online articles published from August 2016 to May 2017.

1. *Bresciaoggi* [Internet]. Meningite alla Gmg: «Qui nessun rischio» 2016 Aug 3. Available from: <http://www.bresciaoggi.it/territori/citt%C3%A0/meningite-alla-gmg-qui-nessun-rischio-1.5043678> Accessed 23 July 2019
2. *Bresciaoggi* [Internet]. 2016 Oct 22. Available from: <http://www.bresciaoggi.it/territori/citt%C3%A0/1.5223233> Accessed 23 July 2019
3. *Bresciaoggi.* [Internet]. Morte in ospedale: sospetta meningite. 2016 Dec 11. Available from: <http://www.bresciaoggi.it/territori/citt%C3%A0/morte-in-ospedale-sospetta-meningite-1.5344283> Accessed 23 July 2019
4. *Bresciaoggi.* [Internet]. Morte improvvisa, c’è l’ombra meningite. 2016 Dec 11. Available from: <http://www.bresciaoggi.it/territori/morte-improvvisa-c-%C3%A8-l-ombra-meningite-1.5344313> Accessed 23 July 2019
5. *Bresciaoggi*. [Internet]. Infezione fulminante a Cologne. La risposta affidata alle analisi. 2016 Dec 12. Available from: <http://www.bresciaoggi.it/territori/infezione-fulminante-a-colognela-risposta-affidata-alle-analisi-1.5346401> Accessed 23 July 2019
6. *Bresciaoggi.* [Internet]. I test confermano: Renato Goffi ucciso dalla meningite. 2016 Dec 13. Available from: <http://www.bresciaoggi.it/i-test-confermano-renato-goffi-ucciso-dalla-meningite-1.5349038> Accessed 23 July 2019
7. *Bresciaoggi.* [Internet]. Un lungo corteo per l’ultimo saluto a Renato. 2016 Dec 14. Available from: <http://www.bresciaoggi.it/territori/sebino-franciacorta/un-lungo-corteo-per-l-ultimo-saluto-a-renato-1.5351300> Accessed 23 July 2019
8. *Bresciaoggi*. [Internet]. «Meningite, nessun rischio» 2016 Dec 23. Available from: <http://www.bresciaoggi.it/territori/citt%C3%A0/meningite-nessun-rischio-1.5373313> Accessed 23 July 2019
9. *Bresciaoggi.* [Internet]. «Vaccinarsi per proteggere noi stessi e l’intera collettività». 2016 Dec 23. Available from: <http://www.bresciaoggi.it/territori/citt%C3%A0/vaccinarsi-per-proteggerenoi-stessi-e-l-intera-collettivit%C3%A0-1.5373350> Accessed 23 July 2019
10. *Bresciaoggi.* [Internet]. Sospetta meningite a Bagnolo: scatta la profilassi. 2017 Jan 3. Available from: <http://www.bresciaoggi.it/territori/bassa/sospetta-meningite-a-bagnolo-scatta-la-profilassi-1.5391114> Accessed 23 July 2019
11. *Bresciaoggi.* [Internet]. Allarme meningite: da lunedì vaccinazioni «libere» per tutti. 2017 Jan 4. Available from: <http://www.bresciaoggi.it/territori/citt%C3%A0/allarme-meningite-da-luned%C3%ACvaccinazioni-libere-per-tutti-1.5393393> Accessed 23 July 2019
12. *Bresciaoggi.* [Internet]. Meningite, parte la campagna regionale per la vaccinazione. 2017 Jan 4. Available from: <http://www.bresciaoggi.it/territori/citt%C3%A0/meningite-parte-la-campagna-regionale-per-la-vaccinazione-1.5393432> Accessed 23 July 2019
13. *Bresciaoggi.* [Internet]. «Meningite, la diagnosi rapida è decisiva» 2017 Jan 4. Available from: <http://www.bresciaoggi.it/home/altri/interviste/meningite-la-diagnosi-rapida-%C3%A8-decisiva-1.5393527> Accessed 23July 2019
14. *Bresciaoggi*. [Internet]. Vaccino per la meningite centinaia di prenotazioni. 2017 Jan 10. Available from: <http://www.bresciaoggi.it/territori/citt%C3%A0/vaccino-per-la-meningitecentinaia-di-prenotazioni-1.5405851> Accessed 23 July 2019
15. *Bresciaoggi.* [Internet]. Meningite, per il vaccino è boom di richieste. 2017 Jan 10. Available from: <http://www.bresciaoggi.it/territori/citt%C3%A0/meningite-per-il-vaccino-%C3%A8-boom-di-richieste-1.5405871> Accessed 23 July 2019
16. *Bresciaoggi.* [Internet]. Bimba morta di meningite: medico indagato. 2017 Jan 12. Available from: <http://www.bresciaoggi.it/home/2.3216?q=meningite&page=7> Accessed 28 June 2018
17. *Bresciaoggi*. [Internet]. Meningite: muore al Civile bambina di cinque anni. 2017 Jan 13. Available from: <http://www.bresciaoggi.it/territori/citt%C3%A0/meningite-muore-al-civile-bambina-di-cinque-anni-1.5413740> Accessed 23 July 2019
18. *Bresciaoggi.* [Internet]. Meningite, muore a cinque anni al Civile. 2017 Jan 13. Available from: <http://www.bresciaoggi.it/territori/citt%C3%A0/meningite-muore-a-cinque-anni-al-civile-1.5413758> Accessed 23 July 2019
19. *Bresciaoggi*. [Internet]. Bambina muore di meningite al Civile. 2017 Jan 13. Available from: <http://www.bresciaoggi.it/home/2.3216?q=meningite&page=6> Accessed 28 June 2018
20. *Bresciaoggi.* [Internet]. Vaccinazioni, in provincial quasi 4mila prenotazioni. 2017 Jan 14. Available from: <http://www.bresciaoggi.it/territori/citt%C3%A0/vaccinazioni-in-provinciaquasi-4mila-prenotazioni-1.5416224> Accessed 23 July 2019
21. *Bresciaoggi.* [Internet]. Inzino, il dolore e lo sgomento. 2017 Jan 14. Available from: <http://www.bresciaoggi.it/territori/citt%C3%A0/inzino-il-dolore-e-lo-sgomento-1.5416226> Accessed 23 July 2019
22. *Bresciaoggi.* [Internet]. Meningite, pm al lavoro. Un medico è indagato. 2017 Jan 14. Available from: <http://www.bresciaoggi.it/territori/citt%C3%A0/meningite-pm-al-lavoroun-medico-%C3%A8-indagato-1.5416247> Accessed 23 July 2019
23. *Bresciaoggi*. [Internet]. Meningite, insediata la commissione dell’Ats. 2017 Jan 15. Available from: <http://www.bresciaoggi.it/territori/citt%C3%A0/meningite-insediata-la-commissione-dell-ats-1.5418330> Accessed 23 July 2019
24. *Bresciaoggi*. [Internet]. Meningite, vaccinazioni iniziate con già 1.300 richieste all’Asst. 2017 Jan 17. Available from: <http://www.bresciaoggi.it/territori/citt%C3%A0/meningite-vaccinazioni-iniziate-con-gi%C3%A0-1-300-richieste-all-asst-1.5422720> Accessed 23 July 2019
25. *Bresciaoggi.* [Internet]. Morta per la meningite: sono 8 i medici indagati. 2017 Jan 18. Available from: <http://www.bresciaoggi.it/territori/citt%C3%A0/morta-per-la-meningite-sono-8-i-medici-indagati-1.5425429> Accessed 23 July 2019
26. *Bresciaoggi.* [Internet]. Bimba morta di meningite: otto indagati. 2017 Jan 18. Available from: <http://www.bresciaoggi.it/territori/citt%C3%A0/bimba-morta-di-meningite-otto-indagati-1.5425450> Accessed 23 July 2019
27. *Bresciaoggi*. [Internet]. Adulti e bambini in coda per la vaccinazione: «Una precauzione in più» 2017 Jan 18. Available from: <http://www.bresciaoggi.it/territori/citt%C3%A0/adulti-e-bambini-in-coda-per-la-vaccinazione-una-precauzione-in-pi%C3%B9-1.5425539> Accessed 23 July 2019
28. *Bresciaoggi.* [Internet]. Rientra l’allarme al Civile per un altro caso sospetto. 2017 Feb 21. Available from: <http://www.bresciaoggi.it/rientra-l-allarme-al-civile-per-un-altro-caso-sospetto-1.5509123> Accessed 23 July 2019
29. *Bresciaoggi.* [Internet]. Meningite, uno studente in rianimazione. 2017 Feb 21. Available from: <http://www.bresciaoggi.it/territori/meningite-uno-studente-in-rianimazione-1.5509191> Accessed 23 July 2019
30. *Bresciaoggi.* [Internet]. Diagnosi precoce del virus: la rete dell’Ats mobilitata per monitorare il territorio. 2017 Feb 21. Available from: <http://www.bresciaoggi.it/diagnosi-precoce-del-virus-la-rete-dell-ats-mobilitata-per-monitorare-il-territorio-1.5509271> Accessed 23 July 2019
31. *Bresciaoggi.* [Internet]. Meningite: 22 mila prenotazioni. 2017 Feb 22. Available from: <http://www.bresciaoggi.it/territori/hinterland/meningite-22-mila-prenotazioni-1.5511588> Accessed 23 July 2019
32. *Bresciaoggi.* [Internet]. Tavola rotonda a Milano Rolfi chiama i pediatri. 2017 Feb 22. Available from: <http://www.bresciaoggi.it/territori/hinterland/tavola-rotonda-a-milanorolfi-chiama-i-pediatri-1.5511590> Accessed 23 July 2019
33. *Bresciaoggi.* [Internet]. Stazionario lo studente colpito. 2017 Feb 22. Available from: <http://www.bresciaoggi.it/territori/bassa/stazionario-lo-studente-colpito-1.5511594> Accessed 23 July 2019
34. *Bresciaoggi*. [Internet]. Le vaccinazioni volano, l’attesa oltre 2anni. 2017 Feb 22. Available from: <http://www.bresciaoggi.it/territori/le-vaccinazioni-volano-l-attesa-oltre-2anni-1.5511800> Accessed 23 July 2019
35. *Bresciaoggi*. [Internet]. Istituto superiore della Sanità sul caso di meningite. 2017 Feb 23. Available from: <http://www.bresciaoggi.it/territori/bassa/istituto-superiore-della-sanit%C3%A0-sul-caso-di-meningite-1.5514270> Accessed 23 July 2019
36. *Bresciaoggi.* [Internet]. Bambina muore in città di meningite: 22 indagati. 2017 Feb 28. Available from: <http://www.bresciaoggi.it/territori/citt%C3%A0/bambina-muore-in-citt%C3%A0di-meningite-22-indagati-1.5526456> Accessed 23 July 2019
37. *Bresciaoggi.* [Internet]. Muore di meningite al Civile. In due ospedali 22 indagati. 2017 Feb 28. Available from: <http://www.bresciaoggi.it/territori/citt%C3%A0/muore-di-meningite-al-civile-in-due-ospedali-22-indagati-1.5526477> Accessed 23 July 2019
38. *Corriere della sera.* [Internet]. Meningite alla Gmg, la Diocesi di Brescia: «Qui non ci sono contagi» 2016 Aug 2. Available from: <http://brescia.corriere.it/notizie/cronaca/16_agosto_02/meningite-gmg-diocesi-brescia-qui-non-ci-sono-contagi-cf955fc0-5899-11e6-b011-ed7749260a21.shtml> Accessed 26 July 2019
39. *Corriere della sera.* [Internet]. Meningite alla Gmg, rischio contagio possibile per massimo 10 giorni. 2016 Aug 2. Available from: <http://brescia.corriere.it/notizie/cronaca/16_agosto_02/meningite-gmg-rischio-contagio-possibile-massimo-10-giorni-a1e9cfa4-58c9-11e6-b011-ed7749260a21.shtml> Accessed 26 July 2019
40. *Corriere della sera.* [Internet]. Meningite alla Gmg, scatta la profilassi per 4 ragazzi. 2016 Aug 3. Available from: [http://brescia.corriere.it/notizie/cronaca/16_agosto_03/meningite-gmg-scatta-profilassi-4-ragazzi-09125e2e-5944-11e6-9678-6c5e366d4cd4.shtml Accessed 26 July 2019](http://brescia.corriere.it/notizie/cronaca/16_agosto_03/meningite-gmg-scatta-profilassi-4-ragazzi-09125e2e-5944-11e6-9678-6c5e366d4cd4.shtml%20Accessed%2026%20July%202019)
41. *Corriere della sera.* [Internet]. Uniti nella prevenzione: fare chiarezza sui vaccini. 2016 Aug 4. Available from: <http://brescia.corriere.it/notizie/cronaca/16_agosto_04/chiarezza-vaccini-brescia-copertura-meningite-lucia-monini-e1452536-5a13-11e6-bfed-33aa6b5e1635.shtml> Accessed 26 July 2019
42. *Corriere della sera.* [Internet]. Brescia: allarme medici di famiglia, metà in pensione entro 10 anni. 2016 Aug 8. Available from: <http://brescia.corriere.it/notizie/cronaca/16_agosto_08/brescia-medici-famiglia-base-pensione-eta-media-c983f14a-5d40-11e6-bfed-33aa6b5e1635.shtml> Accessed 26 July 2019
43. *Corriere della sera.* [Internet]. Villaggio Prealpino, caso di meningite in una scuola elementare. 2016 Oct 21. Available from: <http://brescia.corriere.it/notizie/cronaca/16_ottobre_21/villaggio-prealpino-caso-meningite-una-scuola-elementare-452bfd24-97b6-11e6-bd66-b2bce124488b.shtml> Accessed 26 July 2019
44. *Corriere della sera.* [Internet]. Imprenditore di Cologne stroncato dalla meningite: martedì I funerali. 2016 Dec 12. Available from: <http://brescia.corriere.it/notizie/cronaca/16_dicembre_12/imprenditore-cologne-stroncato-meningite-funerali-brescia-8149a240-c040-11e6-84a3-703e0bacaa0c.shtml> Accessed 26 July 2019
45. *Corriere della sera*. [Internet]. A Brescia è incubo Meningite, corsa ai vaccini: vendite record. 2016 Dec 23. Available from: <http://brescia.corriere.it/notizie/cronaca/16_dicembre_23/brescia-paura-meningite-vaccino-farmacia-morti-59970642-c8e3-11e6-bac6-8c33946b31a6.shtml> Accessed 26 July 2019
46. *Corriere della sera.* [Internet]. Meningite, nuovo caso in provincia: 20enne ricoverato in ospedale. 2017 Jan 2. Available from: <http://brescia.corriere.it/notizie/cronaca/17_gennaio_02/meningite-nuovo-caso-provincia-20enne-ricoverato-ospedale-240c7076-d11b-11e6-bd06-82890b12aab1.shtml> Accessed 26 July 2019
47. *Corriere della sera.* [Internet]. Meningite, le informazioni su vaccini e prenotazioni in rete. 2017 Jan 4. Available from: <http://brescia.corriere.it/notizie/cronaca/17_gennaio_04/meningite-informazioni-vaccini-prenotazioni-rete-fdf2f4aa-d255-11e6-af42-cccac9ae7941.shtml> Accessed 26 July 2019
48. *Corriere della sera.* [Internet]. Meningite, Brescia iniziate le prenotazioni online dei vaccini. 2017 Jan 9. Available from: <http://brescia.corriere.it/notizie/cronaca/17_gennaio_09/meningite-brescia-iniziate-prenotazioni-online-vaccini-e9788e96-d65a-11e6-b48b-df5f96e3114a.shtml> Accessed 26 July 2019
49. *Corriere della sera.* [Internet]. Meningite, centinaia di chiamate per le prenotazioni del vaccino. 2017 Jan 9. Available from: <http://brescia.corriere.it/notizie/cronaca/17_gennaio_09/meningite-centinaia-chiamate-le-prenotazioni-vaccino-df78f1ce-d6a0-11e6-b48b-df5f96e3114a.shtml> Accessed 26 July 2019
50. *Corriere della sera.* [Internet]. Meningite, ora il vaccino si prenota anche con la mail. 2017 Jan 11. Available from: <http://brescia.corriere.it/notizie/cronaca/17_gennaio_11/meningite-ora-vaccino-si-prenota-anche-la-mail-e61cb5ac-d7d5-11e6-9dfa-46bea8378d9f.shtml> Accessed 26 July 2019
51. *Corriere della sera.* [Internet]. Meningite a Brescia: morta una bambina di cinque anni. 2017 Jan 12. Available from: <http://brescia.corriere.it/notizie/cronaca/17_gennaio_12/meningite-brescia-morta-bambina-cinque-anni-79f0a2da-d8bb-11e6-97e6-e1e054cdfc34.shtml> Accessed 26 July 2019
52. *Corriere della sera.* [Internet]. Brescia, bimba muore di meningite: era stata dimessa, medico indagato. 2017 Jan 13. Available from: <http://brescia.corriere.it/notizie/cronaca/17_gennaio_13/brescia-bambina-cinque-anni-muore-meningite-non-era-vaccinata-ec5436cc-d963-11e6-9668-96e09f069892.shtml> Accessed 26 July 2019
53. *Corriere della sera.* [Internet]. Bimba muore di meningite, a Brescia è boom di richieste per vaccinarsi. 2017 Jan 14. Available from: <http://brescia.corriere.it/notizie/cronaca/17_gennaio_14/bimba-muore-meningite-brescia-boom-richieste-vaccinarsi-8a26c998-da2f-11e6-817c-c522bb7cbdb6.shtml> Accessed 26 July 2019
54. *Corriere della sera*. [Internet]. Bambina morta per meningite, commissione d’inchiesta al lavoro. 2017 Jan 16. Available from: <http://brescia.corriere.it/notizie/cronaca/17_gennaio_16/brescia-meningite-commissione-d-inchiesta-lavoro-37a96b56-dbc1-11e6-8880-ab80bbeec765.shtml> Accessed 26 July 2019
55. *Corriere della sera.* [Internet]. Bambina morta di meningite, indagati otto medici del Civile. 2017 Jan 17. Available from: <http://brescia.corriere.it/notizie/cronaca/17_gennaio_17/bambina-morta-meningite-indagati-otto-medici-civile-818efd6c-dca7-11e6-8f57-4c08b8d088ab.shtml> Accessed 26 July 2019
56. *Corriere della sera.* [Internet]. Piccola morta di meningite: I medici indagati salgono a otto. 2017 Jan 18. Available from: <http://brescia.corriere.it/notizie/cronaca/17_gennaio_18/meningite-brescia-ospedale-civile-medici-indagati-dd1395b6-dd52-11e6-bc4e-e834b97e9c52.shtml> Accessed 26 July 2019
57. *Corriere della sera.* [Internet]. Tra Brescia e Islamabad: il doppio addio alla piccola morta di meningite. 2017 Jan 19. Available from: <http://brescia.corriere.it/notizie/cronaca/17_gennaio_19/brescia-islamabad-doppio-addio-piccola-morta-meningite-1d3f83e0-de37-11e6-93cd-d08bed2f6059.shtml> Accessed 26 July 2019
58. *Corriere della sera*. [Internet]. Psicosi meningite, per il vaccino una lista d’attesa di due anni. 2017 Feb 8. Available from: <http://brescia.corriere.it/notizie/cronaca/17_febbraio_08/psicosi-meningite-il-vaccino-lista-d-attesa-due-anni-bfb2db60-edf3-11e6-a862-71d7d0cd9644.shtml> Accessed 26 July 2019
59. *Corriere della sera*. [Internet]. Meningite: 19enne ricoverato a Brescia, profilassi per 50 persone. 2017 Feb 21. Available from: <http://brescia.corriere.it/notizie/cronaca/17_febbraio_21/meningite-19enne-ricoverato-brescia-profilassi-50-persone-8cf936cc-f84f-11e6-b362-d2e82fbd3a4a.shtml> Accessed 26 July 2019
60. *Corriere della sera.* [Internet]. Brescia: muore di meningite a 4 anni, indagati 22 dipendenti dell’ospedale. 2017 Feb 27. Available from: <http://brescia.corriere.it/notizie/cronaca/17_febbraio_27/brescia-bambina-muore-meningite-all-ospedale-indagati-22-dipendenti-2894b41c-fd0a-11e6-8717-6cdb036394a5.shtml> Accessed 26 July 2019
61. *Corriere della sera.* [Internet]. Meningite: dimesso il ragazzo di 19 anni ricoverato a Manerbio. 2017 Feb 28. Available from: <http://brescia.corriere.it/notizie/cronaca/17_febbraio_28/meningite-dimesso-ragazzo-19-anni-ricoverato-manerbio-e126ba48-fdd2-11e6-8934-cbc72457550a.shtml> Accessed 26 July 2019
62. *Corriere della sera*. [Internet]. A Brescia vaccinazioni in calo per morbillo e papilloma virus. 2017 Apr 26. Available from: <http://brescia.corriere.it/notizie/cronaca/17_aprile_26/brescia-vaccinazioni-calo-morbillo-papilloma-virus-0807f896-2a71-11e7-aac7-9deed828925b.shtml> Accessed 26 July 2019
63. *Corriere della sera*. [Internet]. Morbillo, in tremila senza vaccine. Rischiano di non entrare all’asilo. 2017 May 22. Available from: <http://brescia.corriere.it/notizie/cronaca/17_maggio_22/morbillo-tremila-senza-vaccino-rischiano-non-entrare-all-asilo-fcc6bb76-3eba-11e7-a386-529fb6dcf067.shtml> Accessed 26 July 2019
64. *Il giornale di Brescia*. [Internet]. Meningite alla Gmg, morta una 19enne romana. «Evitare allarmismi». 2016 Aug 2. Available from: <https://www.giornaledibrescia.it/italia-ed-estero/meningite-alla-gmg-morta-una-19enne-romana-evitare-allarmismi-1.3108756> Accessed 23 July 2019
65. *Il giornale di Brescia*. [Internet]. Meningite: «Nessun rischio per i bresciani alla Gmg». 2016 Aug 2. Available from: <https://www.giornaledibrescia.it/brescia-e-hinterland/meningite-nessun-rischio-per-i-bresciani-alla-gmg-1.3108764> Accessed 23 July 2019
66. *Il giornale di Brescia*. [Internet]. Meningite alla GMG, le indicazioni dell'ATS. 2016 Aug 3. Available from: [https://www.giornaledibrescia.it/italia-ed-estero/meningite-alla-gmg-le-indicazioni-dell-ats-1.3108881 Accessed 23 July 2019](https://www.giornaledibrescia.it/italia-ed-estero/meningite-alla-gmg-le-indicazioni-dell-ats-1.3108881%20Accessed%2023%20July%202019)
67. *Il giornale di Brescia.* [Internet]. Caso di meningite, profilassi per 143 alunni della primaria Sauro. 2016 Oct 21. Available from: [https://www.giornaledibrescia.it/brescia-e-hinterland/caso-di-meningite-profilassi-per-143-alunni-della-primaria-sauro-1.3125944 Accessed 23 July 2019](https://www.giornaledibrescia.it/brescia-e-hinterland/caso-di-meningite-profilassi-per-143-alunni-della-primaria-sauro-1.3125944%20Accessed%2023%20July%202019)
68. *Il giornale di Brescia.* [Internet]. Meningite, allarme per i tre casi in Toscana. 2016 Nov 23. Available from: [https://www.giornaledibrescia.it/italia-ed-estero/meningite-allarme-per-i-tre-casi-in-toscana-1.3133192 Accessed 23 July 2019](https://www.giornaledibrescia.it/italia-ed-estero/meningite-allarme-per-i-tre-casi-in-toscana-1.3133192%20Accessed%2023%20July%202019)
69. *Il giornale di Brescia.* [Internet]. Meningite fulminante, muore imprenditore 59enne. 2016 Dec 11. Available from: <https://www.giornaledibrescia.it/sebino-e-franciacorta/meningite-fulminante-muore-imprenditore-59enne-1.3136947> Accessed 23 July 2019
70. *Il giornale di Brescia*. [Internet]. Domani l'addio a Renato Goffi, stroncato dalla meningite. 2016 Dec 28. Available from: <https://www.giornaledibrescia.it/sebino-e-franciacorta/domani-l-addio-a-renato-goffi-stroncato-dalla-meningite-1.3137227> Accessed 23 July 2019
71. *Il giornale di Brescia.* [Internet]. Maestra morta di meningite, autopsia per stabilire il ceppo. 2016 Dec 28. Available from:<https://www.giornaledibrescia.it/italia-ed-estero/maestra-morta-di-meningite-autopsia-per-stabilire-il-ceppo-1.3139858> Accessed 23 July 2019
72. *Il giornale di Brescia.* [Internet]. Meningite: le cose da sapere. 2016 Dec 28. Available from: <https://www.giornaledibrescia.it/italia-ed-estero/meningite-le-cose-da-sapere-1.3139862> Accessed 23 July 2019
73. *Il giornale di Brescia*. [Internet]. Maestra morta di meningite, nessun rischio di contagio. 2016 Dec 29. Available from: <https://www.giornaledibrescia.it/italia-ed-estero/maestra-morta-di-meningite-nessun-rischio-di-contagio-1.3139886> Accessed 23 July 2019
74. *Il giornale di Brescia*. [Internet]. Bimbo di 22 mesi muore stroncato da meningite. 2016 Dec 29. Available from: <https://www.giornaledibrescia.it/italia-ed-estero/bimbo-di-22-mesi-muore-stroncato-da-meningite-1.3140034> Accessed 23 July 2019
75. *Il giornale di Brescia*. [Internet]. Meningite: il virologo risponde. https://www.giornaledibrescia.it/italia-ed-estero/meningite-il-virologo-risponde-1.3140040 Accessed 23 July 2019
76. *Il giornale di Brescia*. [Internet]. Altro sospetto caso di meningite a Napoli: muore 46enne. 2016 Dec 30. Available from: <https://www.giornaledibrescia.it/italia-ed-estero/altro-sospetto-caso-di-meningite-a-napoli-muore-46enne-1.3140152> Accessed 23 July 2019
77. *Il giornale di Brescia*. [Internet]. Meningite, vaccino a costo ridotto da gennaio in Lombardia. 2016 Dec 30. Available from: <https://www.giornaledibrescia.it/italia-ed-estero/meningite-vaccino-a-costo-ridotto-da-gennaio-in-lombardia-1.3140193> Accessed 23 July 2019
78. *Il giornale di Brescia*. [Internet]. Elisoccorso per il bimbo: scatta il (falso) allarme meningite. 2016 Dec 30. Available from: <https://www.giornaledibrescia.it/bassa/elisoccorso-per-il-bimbo-scatta-il-falso-allarme-meningite-1.3140199> Accessed 23 July 2019
79. *Il giornale di Brescia*. [Internet]. Nuovo caso di meningite in Toscana, 20enne in terapia intensiva. 2017 Jan 1. Available from: <https://www.giornaledibrescia.it/italia-ed-estero/nuovo-caso-di-meningite-in-toscana-20enne-in-terapia-intensiva-1.3140499> Accessed 23 July 2019
80. *Il giornale di Brescia*. [Internet]. Meningite, nuovo caso nel Bresciano: 20enne in ospedale. 2017 Jan 2. Available from: <https://www.giornaledibrescia.it/bassa/meningite-nuovo-caso-nel-bresciano-20enne-in-ospedale-1.3140838> Accessed 23 July 2019
81. *Il giornale di Brescia*. [Internet]. Code per vaccinarsi dalla meningite B: primo posto a metà 2018. 2017 Jan 3. Available from: <https://www.giornaledibrescia.it/brescia-e-hinterland/code-per-vaccinarsi-dalla-meningite-b-primo-posto-a-met%C3%A0-2018-1.3140835> Accessed 23 July 2019
82. *Il giornale di Brescia*. [Internet]. La meningite fa paura, ma negli ultimi anni i casi sono in calo. 2017 Jan 3. Available from: <https://www.giornaledibrescia.it/brescia-e-hinterland/la-meningite-fa-paura-ma-negli-ultimi-anni-i-casi-sono-in-calo-1.3140879> Accessed 23 July 2019
83. *Il giornale di Brescia*. [Internet]. Meningite: dal 16 gennaio al via le vaccinazioni. 2017 Jan 3. Available from: <https://www.giornaledibrescia.it/brescia-e-hinterland/meningite-dal-16-gennaio-al-via-le-vaccinazioni-1.3140947> Accessed 23 July 2019
84. *Il giornale di Brescia*. [Internet]. Meningite, il germe che la causa non si propaga oltre i due metri. 2017 Jan 4. Available from: <https://www.giornaledibrescia.it/brescia-e-hinterland/meningite-il-germe-che-la-causa-non-si-propaga-oltre-i-due-metri-1.3140993> Accessed 23 July 2019
85. *Il giornale di Brescia*. [Internet]. «La scienza non è democratica»: Burioni è ormai una star di Fb. 2017 Jan 5. Available from: <https://www.giornaledibrescia.it/italia-ed-estero/la-scienza-non-%C3%A8-democratica-burioni-%C3%A8-ormai-una-star-di-fb-1.3141107> Accessed 23 July 2019
86. *Il giornale di Brescia.* [Internet]. Meningite, il Ministero: «Nessuna carenza di vaccini». 2017 Jan 5. Available from: <https://www.giornaledibrescia.it/italia-ed-estero/meningite-il-ministero-nessuna-carenza-di-vaccini-1.3141273> Accessed 23 July 2019
87. *Il giornale di Brescia*. [Internet]. Meningite: dai primi sintomi alle cure. 2017 Jan 5. Available from: <https://www.giornaledibrescia.it/italia-ed-estero/meningite-dai-primi-sintomi-alle-cure-1.3141275> Accessed 23 July 2019
88. *Il giornale di Brescia*. [Internet]. Meningite, da oggi si può prenotare il vaccino. 2017 Jan 9. Available from: <https://www.giornaledibrescia.it/brescia-e-hinterland/meningite-da-oggi-si-pu%C3%B2-prenotare-il-vaccino-1.3141861> Accessed 23 July 2019
89. *Il giornale di Brescia*. [Internet]. Vaccino contro la meningite, lo farete? Diteci la vostra. 2017 Jan 9. Available from: <https://www.giornaledibrescia.it/brescia-e-hinterland/vaccino-contro-la-meningite-lo-farete-diteci-la-vostra-1.3141897> Accessed 23 July 2019
90. *Il giornale di Brescia*. [Internet]. Meningite, boom di prenotazioni per il vaccino. 2017 Jan 10. Available from: <https://www.giornaledibrescia.it/brescia-e-hinterland/meningite-boom-di-prenotazioni-per-il-vaccino-1.3142066> Accessed 23 July 2019
91. *Il giornale di Brescia*. [Internet]. Meningite, bimba di 5 anni muore al Civile di Brescia. 2017 Jan 12. Available from: <https://www.giornaledibrescia.it/valtrompia-e-lumezzane/meningite-bimba-di-5-anni-muore-al-civile-di-brescia-1.3142311> Accessed 23 July 2019
92. *Il giornale di Brescia*. [Internet]. Meningite: non è emergenza, ma scatta la corsa al vaccino. 2017 Jan 12. Available from: <https://www.giornaledibrescia.it/brescia-e-hinterland/meningite-non-%C3%A8-emergenza-ma-scatta-la-corsa-al-vaccino-1.3142344> Accessed 23 July 2019
93. *Il giornale di Brescia*. [Internet]. Bimba morta per meningite, «verifiche sulle procedure» 2017 Jan 12. Available from: <https://www.giornaledibrescia.it/brescia-e-hinterland/bimba-morta-per-meningite-verifiche-sulle-procedure-1.3142372> Accessed 23 July 2019
94. *Il giornale di Brescia*. [Internet]. Muore a 5 anni per meningite: dimessa dopo le prime cure. 2017 Jan 12. Available from: <https://www.giornaledibrescia.it/valtrompia-e-lumezzane/muore-a-5-anni-per-meningite-dimessa-dopo-le-prime-cure-1.3142395> Accessed 23 July 2019
95. *Il giornale di Brescia*. [Internet]. Bimba morta per meningite, la paura lascia il posto al dolore. 2017 Jan 13. Available from: <https://www.giornaledibrescia.it/valtrompia-e-lumezzane/bimba-morta-per-meningite-la-paura-lascia-il-posto-al-dolore-1.3142442> Accessed 23 July 2019
96. *Il giornale di Brescia*. [Internet]. Bimba morta di meningite, medico indagato per omicidio colposo. 2017 Jan 13. Available from: <https://www.giornaledibrescia.it/brescia-e-hinterland/bimba-morta-di-meningite-medico-indagato-per-omicidio-colposo-1.3142451> Accessed July 2019
97. *Il giornale di Brescia*. [Internet]. Meningite, in tilt il centralino per le prenotazioni. 2017 Jan 14. Available from: [https://www.giornaledibrescia.it/brescia-e-hinterland/meningite-in-tilt-il-centralino-per-le-prenotazioni-1.3142589 Accessed](https://www.giornaledibrescia.it/brescia-e-hinterland/meningite-in-tilt-il-centralino-per-le-prenotazioni-1.3142589%20Accessed) 23 July 2019
98. *Il giornale di Brescia*. [Internet]. Bimba morta per meningite: due funerali per la piccola Malika. 2017 Jan 14. Available from: [https://www.giornaledibrescia.it/valtrompia-e-lumezzane/bimba-morta-per-meningite-due-funerali-per-la-piccola-malika-1.3142761 Accessed 23 July 2019](https://www.giornaledibrescia.it/valtrompia-e-lumezzane/bimba-morta-per-meningite-due-funerali-per-la-piccola-malika-1.3142761%20Accessed%2023%20July%202019)
99. *Il giornale di Brescia*. [Internet]. «Fosse stata ricoverata subito, forse sarebbe ancora con noi» 2017 Jan 15. Available from: <https://www.giornaledibrescia.it/valtrompia-e-lumezzane/fosse-stata-ricoverata-subito-forse-sarebbe-ancora-con-noi-1.3142730> Accessed 23 July 2019
100. *Il giornale di Brescia*. [Internet]. Vaccino contro la meningite, centralini ancora intasati. 2017 Jan 16. Available from: <https://www.giornaledibrescia.it/brescia-e-hinterland/vaccino-contro-la-meningite-centralini-ancora-intasati-1.3143234> Accessed 23 July 2019
101. *Il giornale di Brescia.* [Internet]. Meningite, per chi ha prenotato è scattata l'ora del vaccino. 2017 Jan 17. Available from: <https://www.giornaledibrescia.it/brescia-e-hinterland/meningite-per-chi-ha-prenotato-%C3%A8-scattata-l-ora-del-vaccino-1.3143314> Accessed 23 July 2019
102. *Il giornale di Brescia*. [Internet]. Per la morte di Malika 8 medici indagati. 2017 Jan 17. Available from: <https://www.giornaledibrescia.it/brescia-e-hinterland/per-la-morte-di-malika-8-medici-indagati-1.3143320> Accessed 23 July 2019
103. *Il giornale di Brescia*. [Internet]. Meningite: nuovo numero per prenotare il vaccino. 2017 Jan 17. Available from: <https://www.giornaledibrescia.it/brescia-e-hinterland/meningite-nuovo-numero-per-prenotare-il-vaccino-1.3143353> Accessed 23 July 2019
104. *Il giornale di Brescia*. [Internet]. Meningite: muore in pronto soccorso al Cardarelli. 2017 Jan 18. Available from: <https://www.giornaledibrescia.it/italia-ed-estero/meningite-muore-in-pronto-soccorso-al-cardarelli-1.3143450> Accessed 23 July 2019
105. *Il giornale di Brescia*. [Internet]. Sospetta meningite a Manerbio, 19enne in Rianimazione. 2017 Feb 21. Available from: <https://www.giornaledibrescia.it/bassa/sospetta-meningite-a-manerbio-19enne-in-rianimazione-1.3150735> Accessed 23 July 2019
106. *Il giornale di Brescia*. [Internet]. Colpito da meningite a 19 anni, ora è in condizioni stabili. 2017 Feb 21. Available from: <https://www.giornaledibrescia.it/bassa/colpito-da-meningite-a-19-anni-ora-%C3%A8-in-condizioni-stabili-1.3150834> Accessed 23 July 2019
107. *Il giornale di Brescia*. [Internet]. Meningite, per la vaccinazione si aspetta fino a luglio 2019. 2017 Feb 21. Available from: <https://www.giornaledibrescia.it/brescia-e-hinterland/meningite-per-la-vaccinazione-si-aspetta-fino-a-luglio-2019-1.3150840> Accessed 23 July 2019
108. *Il giornale di Brescia*. [Internet]. Meningite, il 19enne migliora ancora. Da stabilire il ceppo. 2017 Feb 23. Available from: <https://www.giornaledibrescia.it/bassa/meningite-il-19enne-migliora-ancora-da-stabilire-il-ceppo-1.3151097>. Accessed 23 July 2019
109. *Il giornale di Brescia*. [Internet]. Meningite, bimba di Cremona morta al Civile: 22 medici indagati. 2017 Feb 28. Available from: <https://www.giornaledibrescia.it/brescia-e-hinterland/meningite-bimba-di-cremona-morta-al-civile-22-medici-indagati-1.3152194> Accessed 23 July 2019
110. *Il giornale di Brescia*. [Internet]. Meningite, sta bene ed è tornato a casa lo studente di Manerbio. 2017 Feb 28. Available from: <https://www.giornaledibrescia.it/bassa/meningite-sta-bene-ed-%C3%A8-tornato-a-casa-lo-studente-di-manerbio-1.3152298> Accessed 23 July 2019
111. *Il giornale di Brescia*. [Internet]. Vaccinazioni: per i neonati pretermine nessuna differenza. 2017 Apr 24. Available from: <https://www.giornaledibrescia.it/rubriche/salute-e-benessere/medicina/vaccinazioni-per-i-neonati-pretermine-nessuna-differenza-1.3165657> Accessed 23 July 2019
112. *Il giornale di Brescia*. [Internet]. Dal morbillo alla meningite: via libera ai 12 vaccini obbligatori. 2017 May 19. Available from: https://www.. giornaledibrescia.it/italia-ed-estero/da-morbillo-a-meningite-via-libera-ai-12-vaccini-obbligatori-1.3174027 Accessed 28 June 2018
